# The Role of miRNAs in Angiogenesis, Invasion and Metabolism and Their Therapeutic Implications in Gliomas

**DOI:** 10.3390/cancers9070085

**Published:** 2017-07-10

**Authors:** Sasha Beyer, Jessica Fleming, Wei Meng, Rajbir Singh, S. Jaharul Haque, Arnab Chakravarti

**Affiliations:** Department of Radiation Oncology, the Ohio State University Comprehensive Cancer Center & Arthur, G. James Cancer Hospital, Columbus, OH 43012, USA; sasha.beyer@osumc.edu (S.B.); jessica.fleming@osumc.edu (J.F.); wei.meng@osumc.edu (W.M.); rajbir.singh@osumc.edu (R.S.); saikh.haque@osumc.edu (S.J.H.)

**Keywords:** glioma, glioblastoma, miRNA, angiogenesis, invasion, cell metabolism

## Abstract

MicroRNAs (miRNAs) are small, non-coding, endogenous RNA molecules that function in gene silencing by post-transcriptional regulation of gene expression. The dysregulation of miRNA plays a pivotal role in cancer tumorigenesis, including the development and progression of gliomas. Their small size, stability and ability to target multiple oncogenes have simultaneously distinguished miRNAs as attractive candidates for biomarkers and novel therapeutic targets for glioma patients. In this review, we summarize the most frequently cited miRNAs known to contribute to gliomagenesis and progression by regulating the defining hallmarks of gliomas, including angiogenesis, invasion, and cell metabolism. We also discuss their promising potential as prognostic and predictive biomarkers and novel therapeutic targets, in addition to the challenges that must be overcome before their translation from bench to bedside.

## 1. Introduction

MicroRNAs (miRNAs) are small, non-coding, endogenous RNA molecules approximately 14–22 nucleotides in length, which function in gene silencing by post-transcriptional regulation of protein expression. miRNAs hybridize to the 3′-untranslated regions (UTRs) of target mRNAs, and either inhibit translation or promote degradation of transcripts. Since miRNAs are not a perfect complement of their target mRNA, they have the ability to target tens to thousands of genes whose protein products function in various signaling pathways. This combined effect on multiple target genes may dysregulate multiple pathways and networks in carcinogenesis, which brings promise to miRNAs as effective therapeutic targets.

Based on their expression levels and major target oncogenes (or tumor suppressor genes), miRNAs may act as oncogenic miRNAs (onco-miRs) (or tumor suppressors miRNAs) in cancer development and progression [1]. Onco-miRs are generally upregulated in cancer, and contribute to tumorigenesis by silencing tumor suppressor genes, while tumor suppressor miRNAs are generally down-regulated in cancers, and function by silencing target oncogenes involved in tumorigenesis [1]. Given their critical roles in carcinogenesis, miRNAs themselves are also subject to regulatory controls at both post-transcriptional and epigenetic levels. The mutations in epigenetic modifiers, such as isocitrate dehydrogenase (IDH1/2), telomerase reverse transcriptase (TERT), and alpha-thalassemia/mental retardation syndrome X-linked (ATRX), which lead to global changes in the epigenome, are common drivers of gliomagenesis [2,3]. The roles that these mutations play in miRNA dysregulation and glioma development are poorly understood.

In this review, we discuss dysregulated miRNAs and their involvement in glioma development and progression. More specifically, we have performed a comprehensive review of miRNAs targeting a number of hallmarks of malignant gliomas, which include angiogenesis, invasion, and altered metabolism. To this end, we have identified and discussed the most frequently cited miRNAs involved in angiogenesis, invasion, and glioma cell metabolism. Therapeutic implications for these miRNAs and the challenges for their clinical translation are also discussed.

## 2. miRNA Biogenesis

**Figure 1:**
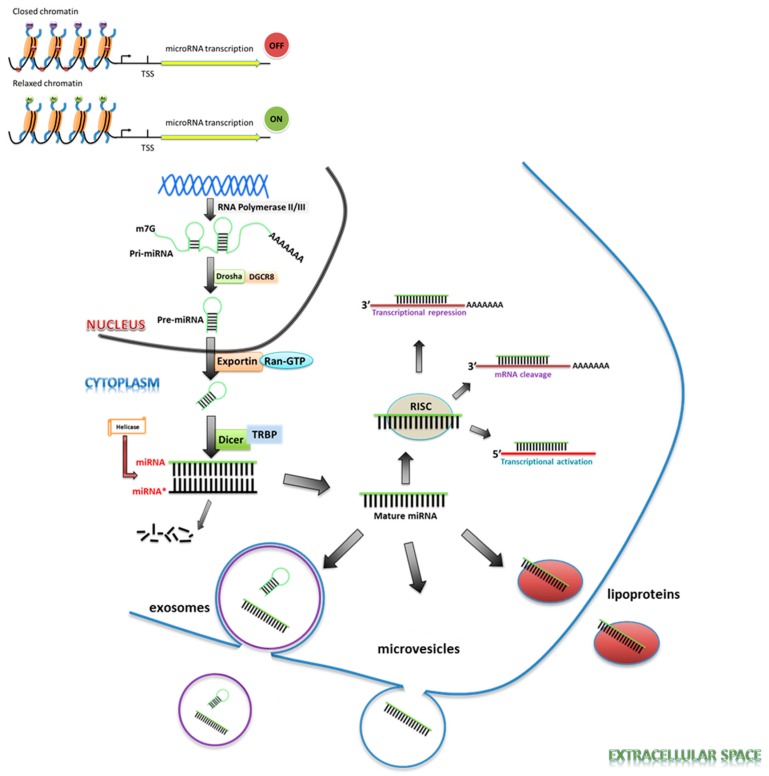
Biogenesis and regulation of microRNA (miRNA). The transcription of miRNA is regulated by epigenetic mechanisms in addition to other levels. The interplay between DNA hypermethylation and H3 lysine 9 and 20 trimethylation can form condensed and inactive chromatin structures to suppress the transcription of miRNA genes. On the other hand, DNA hypomethylation and loss of H3 lysine 9 and 20 trimethylation will reverse chromatin condensation, resulting in transcription of miRNA genes. The miRNA genes are transcribed by Polymerase II/III into a structure that folds back to form a hairpin loop. This structure is known as a primary-miRNA (pri-miRNA), which is capped and polyadenylated. The pri-miRNA is then recognized and cleaved by a nuclear protein, in complex with Dgcr8/Drosha, to form precursor miRNA (pre-miRNA) in the nucleus, which is transported into the cytoplasm by Exportin-5. The exported pre-miRNA is cleaved by RNAse III enzyme Dicer, in association with the human immunodeficiency virus transactivating response RNA-binding protein (TRBP). The product is an imperfect miRNA: miRNA duplex about 22 nucleotides in length. The duplex is unwound by helicase, after which the strand corresponding to the mature miRNA is loaded onto the RNA-induced silencing complex (RISC). Based on the target and complementarity, mature miRNAs can bind either the 3′-untranslated region of target mRNAs and subsequently block their translation and/or result in mRNA cleavage/degradation or, alternatively, bind to the 5′-UTR region and lead to translational activation.

The generation of mature miRNAs is a multi-step process. Biogenesis starts with the initial transcription of miRNA genes by RNA polymerase II or, alternatively, by RNA polymerase III as co-transcripts with neighboring repetitive elements [4,5]. The initial miRNA transcript is several hundred nucleotides long, capped, polyadenylated, and contains an imperfectly double-stranded region within a hairpin loop. The 5′ and 3′ ends of miRNAs’ primary transcripts are processed by the ribonuclease (RNase) III Drosha-DGCR8 nuclear complex, in an endoribonucleolytic cleavage, into the hairpin structure precursor miRNA of less than a hundred nucleotides. The resulting pre-miRNA is then transported through the nuclear pore into the cytoplasm by exportin-5, in complex with Ran and GTP. Once in the cytoplasm, the pre-miRNA is further cleaved by the RNase enzyme DICER1, an Argonaute protein, and either TARBP2 or PRKRA into double-stranded miRNAs, with protruding single-stranded 3′ ends of 2–3 nucleotides. The two strands are separated by helicases, and the mature strand incorporates into the RNA-induced silencing complex (RISC) [5,6,7]. Here, the passenger strand is degraded and the guide strand is targeted to specific mRNAs (Figure 1). The selection of the strand that can enter the RISC and become the predominant product depends on several factors, including thermodynamic stability, base pairing, and the position of the stem-loop [8].

Typically, mature miRNAs regulate gene expression through sequence-specific binding to the 3′-UTR of an mRNA, though several lines of evidence indicate that miRNAs can also bind to 5′-UTR or other regions of target mRNAs. The seed sequence, located 2–7 nucleotides from the 5′ end of the miRNA, most commonly determines the specific mRNA targets that it binds to. The miRNA-mRNA interaction usually causes translational repression and/or mRNA cleavage, and may reduce the final protein level. Alternatively, miRNAs are also known to increase the translation of a target mRNA by recruiting protein complexes to the AU-rich elements of the mRNA, or indirectly by de-repressing the mRNA translation via interaction with proteins that block the target gene translation [5,9].

While the majority of miRNAs remain intracellular, there is a small population of miRNAs termed cell-free miRNAs or circulating miRNAs that are ejected into the circulation. Alternatively, miRNAs can also be packaged into multivesicular bodies (MVBs) and released into the extracellular environment as exosomes. Following their release, the circulating miRNAs are taken up by the recipient cells in which they execute specific functions, or they may also act as hormones, triggering a receptor-mediated response in a different cell or tissue [10,11]. miRNA biogenesis is therefore regulated at multiple levels by multiple mechanisms and, not surprisingly, each step of miRNA generation and function may be targeted for therapeutics [12].

## 3. miRNA Regulation

The expression of miRNAs is controlled at multiple levels, including the previously discussed steps of miRNA transcription, nuclear exportation, Drosha/Dicer processing, and other potential post-transcriptional modifications [13,14]. Until recently, little was known about the epigenetic regulation of miRNAs. The transcription and splicing of miRNAs are regulated by DNA methylation and histone modifications. When a miRNA or its promoter is located near a CpG island that is methylated, expression of the miRNA is repressed. Histone modifications also regulate miRNA expression. Hypermethylation of DNA and methylation of lysines 9 and 20 of histone H3 form condensed and inactive chromatin, resulting in gene silencing, whereas histone acetylation promotes active transcription of miRNAs [15,16,17,18,19]. Further investigations into the regulation of miRNA expression by the “histone code” will reveal the roles of epigenetic regulators in miRNA expression.

## 4. Gliomas

Gliomas are the most common (~80%) primary tumors of the human central nervous system [20]. Despite recent advances in surgery, chemotherapy, and radiation, prognosis remains poor for many of these patients. Gliomas have traditionally been diagnosed and treated based on their histopathological grade (I through IV) defined by the World Health Organization (WHO) [21]. This grading system was based on morphologic features of tumor tissues, including atypia, mitosis, endothelial proliferation, and necrosis. However, histopathological grading often produced inconsistent clinical outcomes owing to inter-observer variability [22,23,24,25]. To overcome this limitation, the WHO recently reclassified malignant gliomas by integrating molecular biomarkers, including IDH1/2, ATRX, TERT, TP53, CI, and FUBP1 genes, and co-deletion of 1p and 19q chromosome arms [23,24,25].

Lower-grade gliomas (LGGs, grades II and III) and glioblastomas (grade IV) have been redefined by the presence or absence of the IDH1/2 mutation [23,26,27]. Notably, patients with gliomas harboring IDH1/2 mutations have an overall survival (OS) benefit over patients without an IDH1/2 mutation [28,29]. The underlying biology accounting for these differences in clinical response remains elusive. Mutations in IDH1 are shown to induce the accumulation of methylated DNA via inhibition of DNA demethylation which, in turn, causes global promoter methylation and gene silencing [3]. The specific changes in gene expression that result from these epigenetic changes are uncertain; however, they likely include changes in miRNA expression. Interestingly, Cheng et al. found that IDH1/2 mutation status in lower grade gliomas has more impact on miRNA expression profiles than other genomic changes. Moreover, miRNA profiles play a more significant prognostic role in IDH mutant tumors than IDH WT tumors, as evident from the identification of a four-miRNA risk classifier (miR-10b, miR-130b, miR-1304, and miR-302b) in IDH mutant patients [30].

## 5. Defining Hallmarks of Gliomas

Angiogenesis, invasion, and altered cell metabolism are defining hallmarks of gliomas that contribute to gliomagenesis and progression, and ultimately lead to their poor prognostic outcomes. In the following section, we discuss some of the validated miRNAs involved in each of these glioma traits and highlight those found to be most frequently cited in the literature.

### 5.1. Angiogenesis

Angiogenesis and necrosis distinguish high grade glioblastomas from low grade gliomas and contribute to the more aggressive phenotype of glioblastomas [31,32]. Angiogenesis is the formation of new blood vessels by the remodeling of pre-existing blood vessels. This neovascularization allows for increased oxygen and nutrients to be distributed to rapidly dividing tumor cells. As the metabolic demands of the increasing tumor mass outweigh the oxygen supply of the existing vasculature, hypoxic tumor microenvironments develop, which induce the secretion of pro-angiogenic factors, such as vascular endothelial growth factor (VEGF). Increased VEGF and other pro-angiogenic factor levels, in turn, lead to endothelial cell proliferation and the formation of leaky blood vessels. This newly formed abnormal vasculature does not allow for the effective delivery of oxygen and chemotherapy, and therefore further promotes hypoxia and treatment resistance [32,33,34]. Considerable research has been directed toward the development of novel therapies to target the angiogenesis pathway, with the goal of preventing glioma progression and treatment resistance. Some of the most commonly cited miRNAs involved in angiogenesis in gliomas are highlighted below with additional “angio-miRs”, discussed in Table 1.

#### 5.1.1. miR-296

miR-296 is one of the most well-studied miRNAs known to promote angiogenesis [35,36,37,38,39,40]. Wurdinger et al. have shown that VEGF is sufficient to induce miR-296 expression in glioma cells in vitro [41]. miR-296 targets and decreases expression of hepatocyte growth factor-regulated tyrosine kinase substrate (HGS). Since HGS degrades VEGF and platelet-derived growth factor (PDGF) receptors, reduced levels of HGS by miR-296 further promotes angiogenesis [42]. Moreover, this study also confirms that miR-296 promotes angiogenesis in a tumor xenograft model in vivo [41]. In summary, miR-296 is the most widely reported “angio-miR” in gliomas, and its role in tumor development and progression has been confirmed both in vitro and in vivo [41].

#### 5.1.2. miR-7

Babae et al. recently identified miR-7 as an inhibitor of angiogenesis, via a high-throughput screening assay for miRNAs that regulates endothelial cell (EC) growth, and confirmed that miR-7 reduced angiogenesis and tumor cell proliferation both in vitro and in U-87 xenograft model [43]. MiR-7 targets both EGFR and PI3K signaling pathways [35,44,45]. This study also identifies *O*-linked β-*N*-acetylglucosamine transferase (OGT), an enzyme that adds *O*-linked β-*N*-acetylglucosamine (*O*-GlcNAc) moieties to various nuclear and cytosolic proteins, as another miR-7 target. miR-7-mediated silencing of OGT leads to degradation of transcription factors involved in VEGFR2 expression which, in turn, down-regulates VEGFR2 and angiogenesis [43].

### 5.2. Invasion

The highly invasive nature of gliomas is a major contributor to the poor prognosis and treatment resistance in glioma patients. Unlike many other cancer types, gliomas rarely metastasize through the vasculature or lymphatics to organs outside of the brain, but rather infiltrate through the parenchyma of the brain. The reason for this is uncertain, but may be due to limitations posed by the blood-brain barrier or a necessary neuron-specific microenvironment within the brain [49]. A major obstacle to curing gliomas involves the infiltrating tumor cells and their ability to successfully evade surgery and radiation therapies. It is nearly impossible to completely remove these diffusely infiltrating cells by surgical resection [33,50]. The mechanisms of invasion in gliomas are poorly understood, and a better understanding of these mechanisms is necessary for the development of more effective therapies.

Invading glioma cells tend to develop a mesenchymal phenotype and migrate along the blood vessels and white matter tracts within the brain. These migrating glioma cells mimic the migration of early progenitor cells during nervous system development, a process called epithelial-mesenchymal transition (EMT). The process of tumor cell invasion involves detachment of the invading cell from the primary tumor mass, adhesion to the extracellular matrix (ECM), and finally degradation and detachment of the trailing end of the cell from the ECM [49]. Cytoskeletal changes provide the main contractile force to facilitate cell migration. One of the most common signaling pathways involved in cell migration and invasion is the hepatocyte growth factor (HGF) and its receptor, c-MET [51], which are discussed later in this section as targets of miRNAs. The most frequently cited miRNAs that target glioma invasion are discussed below, and others are outlined in Table 2.

#### 5.2.1. miR-21

miR-21 was the first miRNA to be discovered in glioblastomas in 2005, and is also one of the most well-studied onco-miRs to date [35,36,37,38,39,45,52,53,54,55]. miR-21 is up-regulated in gliomas compared to normal brain tissue. Among other cellular functions, it promotes invasion in gliomas by direct down-regulation of inhibitors of matrix metalloproteases (MMPs), proteolytic enzymes that degrade the extracellular matrix. Specific inhibitors of MMPs that are targeted by miR-21 include reversion-inducing-cysteine-rich protein with kazal motifs (RECK), myristoylated alanine-rich C-kinase substrate protein (MARCKS), and tissue inhibitors of metalloproteases 3 (TIMP3), thus leading to activation of MMPs and invasion [56]. Gabriely et al. have shown that inhibition of miR-21 leads to elevation of RECK and TIMP3 expression, and therefore decreases MMPs and invasion in glioma cells lines in vitro as well as a U87 glioma xenograft model in vivo [57].

#### 5.2.2. miR-34a

Multiple studies have shown that miR-34a is down-regulated in glioblastoma tissue, compared to normal tissues [58,59,60]. Among other cellular functions, such as cell proliferation and survival, miR-34a decreases invasion in glioblastoma cell lines, in part by targeting HGF/c-Met and Notch1/2 signaling [58]. Interestingly, a Phase I trial involving a miRNA-34a mimic was initiated in 2013 for liver cancer, lymphoma, small cell lung cancer, and melanoma patients; however, the trial was closed early due to adverse events [61,62,63]. We discuss the possibility of miR-34a as a potential therapeutic target in a later section.

#### 5.2.3. miR-10b

miR-10b is significantly up-regulated in glioblastomas compared to normal brain tissue [64]. Among other oncogenic roles, miR-10b promotes cell invasion in gliomas as well as in other malignancies [35,36,37,38,39,45,52,54]. A direct target of miR-10b that is likely involved in glioma invasion includes homeobox D10 (HOXD10), which negatively regulates uPAR and RhoC invasion signaling [65,66]. Sasayama et al. have shown that expression levels of HOXD10 are inversely correlated with miR-10b, while RhoC and uPAR expression are significantly associated with expression levels of miR-10b. These results suggest that mIR-10b may regulate cell invasion in a RhoC and uPAR-dependent mechanism [65]. Moreover, Lin et al. have validated that miR-10b overexpression increases cell invasion, and its inhibition decreases cell invasion in vitro [67].

### 5.3. Metabolism

Altered cellular metabolism plays an important role in glioma pathogenesis by altering gene expression and, in turn, these gene expression changes may contribute to altered metabolism in cancers. In normal tissues, energy in the form of ATP is primarily generated in mitochondria by the process of oxidative phosphorylation. However, tumor cells have the ability to reprogram their metabolism in order to meet the high energy demands of rapidly growing and proliferating tumor cells. Tumor cells engage in glycolysis even under conditions of adequate oxygenation, a phenomenon known as the Warburg Effect [79,80]. This increased flux toward glycolysis seems to occur early on in tumorigenesis before hypoxic environments develop.

In addition to glucose metabolism, cancer cells also commonly exhibit altered glutamine and lipid metabolism [81,82]. While glutamine is a non-essential amino acid during normal conditions, it is considered to be conditionally essential in times of cellular stress because of increased demands for this amino acid. Cancer cells have increased dependency on glutamine for growth and proliferation, because glutamine is an alternative source of carbon and nitrogen for the biosynthesis of nucleotides and amino acids. Glutamine can be converted to glutamate which is, in turn, converted to alpha-ketoglutarate (α-KG), a TCA cycle intermediate and major source of energy when glucose is scarce. α-KG is also a substrate for enzymes playing a role in cell signaling and epigenetic regulation, including prolyl hydroxylases, histone demethylases, and 5-methylcytosine hydroxylases. Because of its importance in cancer cell metabolism, glutamine deprivation often leads to cancer cell death [83,84,85]. Cancer cells also maintain an increased requirement for lipids and cholesterol by increasing uptake of exogenous lipids, or by increasing their endogenous synthesis [86]. Lipids are required for signal transduction and cell membrane formation in the developing glioma [87]. MiRNAs shown to alter glucose, glutamine, and lipid metabolism in gliomas are discussed in Table 3.

Mutations in IDH and receptor tyrosine kinase pathways, both common genetic mutations in gliomas, are also believed to play a role in metabolic reprogramming. IDH is an enzyme that catalyzes the oxidative decarboxylation of isocitrate to α-KG, generating NADPH from NADP+ [26]. The IDH mutant protein then converts α-KG to 2-hydroxyglutarate. The accumulation of 2-hydroxyglutarate in IDH mutant gliomas inhibits DNA and histone demethylation enzymes, referred to as dioxygenases, and leads to the development of the hypermethylated glioma CpG island phenotype [88]. This metabolic reprogramming has profound effects on the epigenetic regulation of gene expression in gliomas, including the regulation of miRNA. In presence of an IDH mutation, levels of alpha-ketoglutarate are reduced, which therefore results in decreased degradation of hypoxia-inducible factor 1α (HIF-1α) by α-KG-dependent prolylhydroxylases. This ultimately results in increased angiogenesis, invasion, and cell proliferation.

Interestingly, miRNA may directly modulate metabolism by targeting key enzymes and transporters involved in metabolic processes [89]. It has been well-established that PI3K/AKT signaling, a common signaling abnormality in gliomas, is involved in altered lipid metabolism and is a direct target of several miRNAs [90]. As we will discuss in a later section, miR-122 is a major regulator of cholesterol and lipid metabolism in liver cells, and has been translated into a clinical trial for patients with hepatitis C infections [91,92]. Table 3 outlines some of the most commonly cited miRNAs involved in metabolism of gliomas.

#### 5.3.1. miR-153

miR-153 acts as a tumor suppressor that is down-regulated in glioblastoma, compared to normal brain tissues. While miR-153 has been shown to decrease cell proliferation and promote apoptosis in other cancer types in addition to glioblastoma, a recent study showed that this miRNA may target glutaminase, and therefore prevent the utilization of glutamine for alternative energy, carbon, and nitrogen sources in glioma cells [93].

#### 5.3.2. miR-451

miR-451 is a frequently cited miRNA decreased in glioblastoma tissues, compared to normal tissues [36,37,39]. As discussed in Table 3, this miRNA alters cell metabolism and invasion. miR-451 regulates the balance of proliferation and migration in glioma cells, in response to changes in glucose levels, by directly targeting coenzyme A biosynthesis protein 3 (CAB3), which in turn regulates liver kinase B1 (an adenosine monophosphate kinase pathway protein activated in response to metabolic stress) [74,94]. During times of ample glucose levels, miR-451 levels are increased to promote cell growth, whereas glucose deprivation down-regulates miR-451 levels, decreases cell proliferation, and promotes cell survival [94].

## 6. Prognostic and Predictive miRNA Biomarkers

Given the previously discussed roles of miRNAs in glioma development and progression, miRNAs have attracted a great deal of attention as potential biomarkers that may facilitate management decisions for glioma patients. The small size and stability of miRNA in clinical specimens, as well as the efficacy and affordability of miRNA assays, brings promise for the clinical implementation of miRNAs [97]. After the recent addition of prognostic molecular alterations to the WHO classification of gliomas, significant emphasis has been put on identifying miRNA signatures that may help further refine the prognostic classification, as well as define treatment groups.

One widely reported molecular classification system involves glioma subtypes based on an unsupervised clustering of genome-wide mRNA expression, including proneural, neural, classical, and mesenchymal subtypes [98]. However, only the proneural and mesenchymal subtypes have consistently been confirmed in multiple gene expression profiling studies [99]. While gliomas of the proneural subtype tend to more closely resemble normal neurons, and also have better prognoses, gliomas of the mesenchymal subtype have increased invasive and angiogenic potential, in addition to worse prognoses [99]. Interestingly, Ma et al. identified miR-128a, miR-504, miR-124a, and miR-184 to be significantly inversely correlated with mesenchymal marker expression, therefore suggesting that these miRNAs likely suppress mesenchymal signaling in gliomas [100]. Moreover, functional studies inhibiting miR-128 and miR-504 resulted in increased levels of VIM and YKL-40 expression, both mesenchymal genes involved in invasion and angiogenesis. As previously mentioned, other miRNA including miR-21, miR-10b, and miR-221 have also been shown to play a role in mesenchymal glioma migration and invasion [35]. Interestingly, Papagiannakopoulos et al. showed that, in proneural gliomas, miR-128 acts as a tumor suppressor by enhancing neural differentiation and repressing growth, secondary to targeting oncogenic receptor tyrosine kinases [101]. Marziali et al. identified mir-23a, miR-27a, and miR-9-3p as a miRNA signature able to discriminate proneural vs. mesenchymal gliomas in both glioma stem cell cultures and The Cancer Genome Atlas (TCGA) glioblastoma cohort [102]. Furthermore, Li et al. [103] identified prognostic miRNA signatures corresponding to each molecular subtype, including proneural, neural, classical, and mesenchymal; however, only the mesenchymal signature has been validated in an independent cohort.

The utilization of miRNA signatures as independent diagnostic, prognostic, and predictive biomarkers has been extensively reviewed in glioblastoma patients [104]; however, only two prognostic studies in lower grade gliomas have been published to date [30,105]. While individual prognostic miRNAs have been confirmed in many studies, the most robust prognostic models likely consist of miRNA signatures. However, there have been inconsistencies among prognostic miRNA signatures reported in the literature, which may be attributed to small sample sizes, lack of long-term follow up data, utilization of diverse platforms, and normalization techniques. This is particularly true for studies including lower grade gliomas, as these patients are less common and are often lost to follow-up, due to longer survival times. Furthermore, many analyses include patient populations of heterogeneous grade, histology, and treatment modalities.

A recent study reported that glioblastomas (*n* = 35) could be divided into two prognostic subgroups (early death <450 days vs. long-term survival >450 days), based on expression profiles of thirty miRNAs [106]. However, due to the small sample size, this miRNA signature will need to be validated in a larger patient cohort. Additionally, Srinivasan et al. identified a ten miRNA profile that accurately predicts survival among glioblastomas (*n* = 22) from the TCGA database [107]. A more recent analysis using a larger glioblastoma patient population (*n* = 563) from the TCGA cohort identified three miRNAs (miR-222, miR-302d, and miR-646) that independently predict survival among these patients [108]. By also using the TCGA cohort of glioblastoma patients, Hayes et al. generated a risk score based on expression levels of nine miRNAs found to be significantly associated with survival [109]. Intriguingly, miR-222 emerged as a common player in all three studies; however, despite the use of the same TCGA dataset, it was not found to be associated with disease free survival in another study [110]. These inconsistencies across studies emphasize the need for not only utilizing large patient cohorts, but also for standardization and validation of data analyses among studies.

Similar limitations have prevented the identification of miRNA profiles to help predict responses to treatment among glioma patients. Many previously published clinical studies lack appropriate treatment and control arms to determine true predictive markers of treatment response, whereas other studies have patient cohorts too small to allow for the power necessary for interaction tests.

Single predictive miRNAs have been reported; however, to our knowledge there have only been a few reported miRNA signatures that predict treatment response among gliomas. Hayes et al. identified an eight miRNA signature (miR-124a, miR-202, miR-7, miR-222, miR-363, miR-630, miR-663, miR-204) that predicts overall survival only in those glioma patients treated with bevacizumab [104]. Interestingly, increased expression of miR-7, an inhibitor of angiogenesis discussed earlier in this review, was shown to be associated with a poor response to bevacizumab, suggesting that tumors with less angiogenesis will have a worse response to this VEGF-targeted therapy. Additional studies have suggested that individual miRNAs and miRNA signatures are predictive of a treatment response, including those that predict a response to temozolomide (TMZ) in addition to radiation therapy [111] and TMZ alone [112]; however, it remains to be concluded whether or not these are truly predictive biomarkers. Further investigation is imperative to develop and validate clinically relevant miRNA profiles for predicting patients that may or may not respond to treatment.

## 7. miRNA Therapeutics

**Figure 2:**
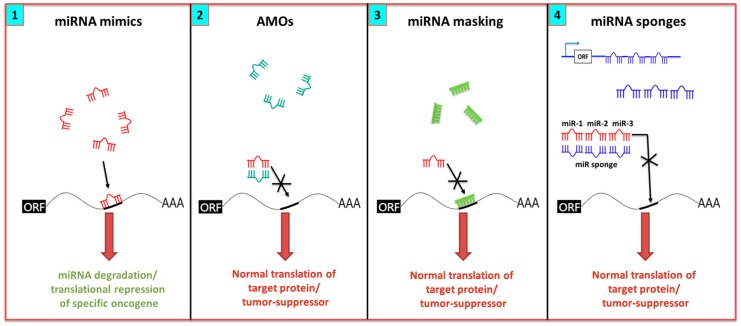
Strategies for manipulation of miRNAs for therapeutic application. Endogenous miRNAs bind to specific regions in 3′-UTR of the mRNA, in order to degrade their target. (**1**) Mimics are synthetic oligonucleotide duplexes that can produce a similar effect, and are used if the expression of miRNA is lost due to the malignant state. (**2**) Oncogenic miRNAs can be downregulated by the use of complimentary miRNAs, known as antagomirs/AMOs (Anti-miRNA oligonucleotides). AMOs (shown in green) bind to their target miRNAs (shown in red), and prevent them from interacting with their target mRNA, thus allowing normal translation. (**3**) The miRNA mask is a construct that is complementary to the mRNA sequence, intended for the binding of a particular miRNA (shown in bright green). The competitive inhibition thus prevents the miRNA binding and allows the translation of mRNA. (**4**) miRNA sponges (shown in blue) are transcripts expressed from strong promoters that display multiple miRNA binding sites. They can be engineered to produce a large quantity of transcripts, in order to target a group/family of miRNAs that share a similar seed sequence.

The potential for miRNAs to simultaneously modulate multiple genes across signaling pathways offers a promising therapeutic approach. They are also attractive candidates, due to their small size, conserved sequences across species, and relative stability. Two common approaches for targeting miRNAs include miRNA mimics and miRNA antagonists, as illustrated in Figure 2. miRNAs with oncogenic function in cancer cells can be reduced by single-stranded anti-miR oligonucleotides [113]. On the other hand, the miRNA tumor suppressor function can be restored by using synthetic double-stranded miRNA that match the corresponding miRNA sequence. While miRNAs offer several advantages as therapeutic targets, we also discuss the challenges associated with their clinical translation, including off-target effects, tissue-specific delivery, complications with cellular uptake, and in vivo instability [114,115].

### 7.1. miRNA Inhibition Strategies

The anti-sense oligonucleotide (anti-miR) is one approach used to ablate miRNA function. It consists of single-stranded RNA oligonucleotides that are complementary and bind to the miRNA, thus preventing the miRNA from binding to its target mRNA. Antagomirs (AMOs) are a class of anti-mir, consisting of chemically-modified single-stranded oligonucleotides that irreversibly and specifically bind to complementary miRNA. Binding of these short oligonucleotides to miRNA prevents miRNA processing or degradation (Figure 2(2)). This approach differs from the classical antisense targeting of mRNAs in the limited range of sites that the miRNA can target [113]. A miRNA mask is comprised of a 22-nucleotide, antisense, single-stranded 2′-*O*-methyl-modified oligoribonucleotide with perfect complementarity to the miRNA target in the 3′-UTR of the mRNA. The binding masks the target site, thereby preventing association with the miRNA and allowing the translation of the mRNA (Figure 2(3)). This approach carries an advantage of annulling the potential off-target effects that may stem from the broad target range of an miRNA [113]. In contrast to AMO, miRNA is not degraded using this approach, therefore the corresponding function of a particular miRNA on other genes remains intact. Locked nucleic acid (LNA) chemistry is a type of antagomir that involves substitution of specific nucleotides with bicyclic RNA analogues in a locked conformation, thus resulting in a higher affinity and better hybridization efficiency [116]. The disadvantages include their limited access to all tissues, requirement for repeated administration in large doses to inhibit miRNAs over long duration, and their tendency to accumulate in the liver [117,118].

miRNA sponges scavenge away the miRNA and prevent it from binding to its mRNA target (Figure 2(4)). A series of RNA sequences that are complementary to the binding site for a specific miRNA are introduced into an expression cassette in the 3′-UTR of a reporter gene. These sites occupy the specific native miRNA and effectively prevent the miRNAs from binding to their mRNA targets [113]. Instead of separately targeting a single miRNA, the approach can scavenge all members at once because it recognizes the same binding sequence [113]. The disadvantage is that sponges use competitive miRNAs that lack chemical modifications, and therefore may suffer from low binding affinity and require a higher concentration for target blocking [119]. Additionally, there is a requirement for strong promoters and the necessity for multiple vector integration [120].

### 7.2. miRNA Replenishment Therapy

Tumor suppressor miRNAs can also be replenished to restore anti-tumor functions by approaches including small molecule modulators, reversal of epigenetic silencing, or introducing miRNA mimics. Small molecule modulators of miRNA function are considered to be potential therapeutic candidates, since they are easily delivered and relatively stable [12]. Epigenetic silencing of miRNA can be reversed by hypomethylating agents such as decitabine or 5-azacytidine. Both agents have been approved for the treatment of myelodisplastic syndromes, and have been shown to re-induce the expression of multiple mRNAs, miRNAs, and other non-coding RNAs. These therapies are gaining attention for improved clinical outcomes in patients with solid tumors, either as a monotherapy or in combination with other therapies [121].

MiRNA mimics are synthetic RNA duplexes in which one strand is identical to the mature miRNA sequence (guide strand) and is designed to “mimic” the function of the endogenous miRNA. The other strand (passenger strand) is often only partially complementary to the guide strand [114,115,122]. The double-stranded structure is required for efficient recognition and loading of the guide strand into the RISC (Figure 2(1)). Care must be taken in the design of such species to eliminate the potential for the passenger strand to act as a new miRNA, and cause unwanted side effects.

### 7.3. Delivery Systems

While many studies have shown promising results for miRNA in vitro, studies with successful miRNA delivery after systemic administration in vivo are limited. Chemical modifications are often required to enhance the stability of miRNAs for delivery, since unmodified miRNAs may be degraded in the blood by nucleases or subsequently cleared via renal secretion or the reticuloendothelial system [123,124,125,126]. In addition to the chemically modified mimics, the use of lenti- and adeno-associated viruses to drive the expression of a given miRNA has been reported by several groups [113,127]. While modified adenovirus or adeno-associated viral vectors may be effective for gene delivery, the issues associated with an immune response to the virus are always a concern, and are discussed in a later section. Therefore, non-viral vectors, which retain biocompatibility, targeting efficacy, and enhanced transfection efficiency, are a more suitable alternative for achieving successful miRNA delivery without the associated side effects.

Oligonucleotides, such as synthetic miRNA mimics and anti-miRNA, can be conjugated or complexed with nanocarriers, thus rendering them more resistant to nuclease degradation. Inorganic nanoparticles (NPs), such as gold (Au), quantum dots, silicon oxide, and iron oxide, are commonly used for oligonucleotide and DNA delivery. Au is an inert element and it does not react with most chemicals, thus making it beneficial for use in living organisms as a potential carrier for oligonucleotides. Recent studies have shown promising results that gold nanoparticles are able to penetrate the blood-brain barrier in vivo [128].

Simple versions of nanoparticles, consisting of PEG-PEI (polyethylenimine) liposomal complexes, have been shown to deliver miRNA mimics, with low immunogenicity and prolonged circulation [127]. Using this approach, many studies have applied PEG-lipid to target liver tumors [12,127]. Cationic lipids and liposomes can form lipoplexes with RNA through electrostatic interactions. In general, lipids used for nucleic acid delivery are composed of a cationic head group and a hydrophobic chain. The choice of the head group and the hydrophobic chain may dramatically affect the transfection efficiency and toxicity level of the lipoplexes. Liposome or nanoparticle-based non-viral delivery system can be used; however, these systems generally suffer from low gene delivery efficiency, especially for in vivo studies [12].

For central nervous system cancers, the blood-brain barrier presents a unique barrier for the delivery of miRNA to target tissues. Recent advancements in drug delivery systems, including cell-penetrating peptides and immunoliposomes, are redesigning therapeutic interventions to help bypass the blood-brain barrier [129].

Even after miRNA therapy is successfully delivered to the tissues of interest, there is still concern for target-specific accumulation and possible side effects associated with supra-physiological dosages of miRNA-associated therapy. Additionally, various mechanical and biological barriers affect the miRNA delivery into the specific target cells, including high interstitial pressure in tumor cells and complexity of the extracellular matrix [48,49,50,51].

## 8. miRNAs in Clinical Trials

A major advantage of miRNAs includes their ability to target multiple genes at once, and therefore they have the potential to effectively address cancer heterogeneity. However, the simultaneous targeting of multiple genes may also lead to unexpected side-effects and unwanted toxicities. The primary requirement for miRNA-associated therapies includes a meticulous selection of candidate miRNA. Ideally, the miRNA should target the desired oncogene(s) with minimal off-target mRNAs. In accordance with these measures, several miRNAs have successfully navigated across the preclinical stage, and are discussed below.

Currently, there are few ongoing Phase I clinical trials utilizing miRNAs as therapeutics in cancer. Unfortunately, there are no Phase I clinical trials for miRNA therapy in gliomas. One miRNA used in the treatment of cancer is miRagen-106, an LNA-modified antisense inhibitor of miRNA-155, for cutaneous T-cell lymphoma [63,130]. Another is an miR-16 mimic therapy for non-small cell lung cancer patients [63,130]. The delivery vehicle for miR-16 is EnGeneIC, a non-living mini-bacteria system that can be modified and used as targeted drug delivery vehicles [63,130].

One of the first tested miRNA therapies for cancer is MRX34, a liposome-formulated synthetic miR-34a mimic (miRNA Therapeutics, Austin, TX, USA). As previously discussed, miR-34a levels are decreased in multiple cancers and function as a tumor suppressor. MRX34 directly inhibits at least 24 different oncogenes, including c-met, Notch, CDK4, and BCL2. Pre-clinical results in multiple mouse models were promising and revealed successful, safe systemic delivery of the miR-34a mimic with no change in cytokine profiles. These studies also demonstrated induction of apoptosis, with an associated tumor response [131,132,133].

A multi-center phase I trial was then initiated in 2013, which involved treatment of primary liver cancer, lymphoma, small cell lung cancer, and melanoma patients with a miR-34a mimic, delivered systemically by intravenous infusion. Substantial evidence of antitumor activity and acceptable safety levels were highlighted in a subset of patients with refractory advanced solid tumors [62]. However, the trial was suspended due to major immune-related adverse events, including severe (Grade 4) cytokine release syndrome. At present, the trigger for these immune reactions is unclear, and pre-clinical trials may have to be repeated [63].

Neurotoxicity induced by miRNA-associated immunomodulation is an important area of investigation. MiRNAs excreted from cancer cells can directly bind to toll-like receptors (TLRs) at the surface of neighboring immune cells, which may lead to activation of the unwarranted signaling pathways in the recipient cells [123,134]. This may result in neurodegeneration, as evident with the let-7b-mediated activation of TLR7 in neurons [135].

Another immuno-toxicity with miRNA-based therapy is the aberrant activation of specific innate immune effector cells, including natural killer (NK) cells via the TLR1-NF-κB pathway. This may affect multiple NK cell functions, including cytokine production, proliferation, and cytotoxicity, all of which may alter the immune response and induce malignant transformation [134,136,137]. Also, it may lead to secretion of inflammatory cytokines and type I interferons (IFNs) by TLRs, based on the structure, sequence, and the delivery system of specific miRNAs, thus affecting the innate and adaptive immune response. This may activate a cascade of events leading to the priming of surrounding immune cells, causing them to become more sensitive to RNA stimulation [123,134,138]. These toxicity issues need to be addressed in order to better understand and prevent immune-related adverse events similar to those that occurred with MRX34.

Another miRNA that has gained an edge in the clinical domain is Miravirsen, which is currently being evaluated in Phase II clinical trials for the treatment of Hepatitis C virus (HCV) infection. Miravirsen is a β-D-oxy-LNA-modified phosphorothioate antisense oligonucleotide that targets miR-122. miR-122 is endogenously expressed in the liver and important in hepatocyte development, differentiation, and metabolism. This miRNA is also involved in replication of Hepatitis C virus RNA when in complex with the Argonaute 2 protein. This miR-122/viral RNA/arogonaute 2 protein complex also helps prevent the nucleolytic degradation of hepatitis C. In the presence of Miravirsen, miR-122 is unable to associate with the complex and the virus cannot replicate [91].

During a recent Phase 2a study for patients with chronic HCV infection, there was a significant dose-dependent decrease in HCV load with Miravirsen therapy. The majority of adverse side effects consisted of Grade I headaches [92]. Miravirsen, in combination with other anti-viral therapies (telaprevir and ribavirin), for the treatment of chronic Hepatitis C virus infection, is currently being studied in a phase II trial [139].

Clinical miRNA trials for other disease types such as type 2 diabetes, non-alcoholic fatty liver disease, and scleroderma also exist. An anti-miRNA therapy utilizing N-Acetylgalactosamine (GalNAc)-conjugated anti-miRNAs for miRNA-103/107 is currently a Phase I trial for patients with type 2 diabetes and non-alcoholic fatty liver diseases. Additionally, scleroderma patients have the opportunity to enroll in a miRNA-29 mimic Phase I trial, which uses a cholesterol-conjugated miRNA duplex-based delivery system [130].

## 9. Conclusions and Perspectives

Although still in its infancy, the translation of miRNAs from the bench to the clinic has the potential to greatly impact personalized medicine for patients with gliomas or other malignancies, either as biomarkers, a monotherapy, or in combination with other treatment modalities. Given their pivotal roles in glioma development and progression, miRNAs have received a great deal of attention as potential therapeutic targets and biomarkers for glioma patients. These are attractive therapeutic candidates, due to their ability to simultaneously modulate multiple genes across signaling pathways, their small size, and their stability. In addition to the therapeutic potential of miRNA, research is currently underway to identify miRNA signatures that may serve as diagnostic, prognostic, and predictive biomarkers for glioma development, progression, and treatment in the clinic. In addition to tissue miRNA biomarkers, miRNAs are readily accessible in biofluids (blood and urine), which may offer an additional, non-invasive source of biomarkers that may not only facilitate diagnosis and prognosis, but may also help determine the best treatment options and monitor treatment response. Furthermore, the low cost, low RNA input requirements and rapid processing of miRNA assays make them efficient and suitable for use in the clinic.

One limitation to the clinical translation of miRNA is identifying of an ideal therapeutic miRNA candidate. An ideal miRNA candidate should have multiple oncogenic targets with limited non-specific targets. The most promising miRNA sequence would likely simultaneously target multiple features of tumor development and progression, including angiogenesis, invasion, and/or cell metabolism. Given that the targets of miRNAs may involve multiple pathways via imperfect matching with 3′-UTRs, off-target gene silencing of tumor suppressor genes may lead to toxicities and/or reduced therapeutic effects. A comprehensive knowledge of the mRNA targets of each miRNA is imperative for avoiding off-target effects and unwanted toxicities.

High-throughput miRNA profiling studies are being utilized to identify novel miRNA and their associated targets. While new strategies for the identification and characterization of targets of individual miRNAs have been developed, limitations still exist. Bioinformatics software use algorithms to predict potential mRNA targets based on the miRNA “seed” sequence, which is typically located on nucleotides 2–7 from the 5′ end of the miRNA [140]. Hundreds of candidate mRNA targets are often generated based on these algorithms, all of which must be confirmed in vitro due to the high likelihood of false positives. While validating these targets in vitro can often be tedious and expensive, confirming targets by an experimental approach is more sensitive and accurate than a computational approach.

Previous studies have identified candidate miRNA by pre-clinical experiments, and while this is valuable, further validation in patient samples needs to be conducted. However, there are limitations involved with screening for miRNA biomarkers in patient samples, including small sample sizes, as well as a lack of long-term follow up and survival data. This is particularly true for lower grade gliomas, compared to glioblastomas, because of their rarity and much longer and variable survival times. Additionally, many of these analyses are performed using heterogeneous patient populations consisting of multiple grades, histologies, and treatment modalities, which create challenges during data analysis and interpretation. Furthermore, glioblastoma has a vastly different genetic make-up from lower grade gliomas, both pathologically and molecularly. While we discuss miRNAs and their involvement in gliomas in general in this review, more work needs to be done to distinguish the miRNAs important for gliomagenesis, and progression in low grade gliomas versus glioblastomas.

The recent failure of the MRX34 trial has provoked the development of a deeper understanding of the barriers and associated toxicities of miRNA-based therapy for cancer. Further pre-clinical and mechanistic studies are imperative for elucidating the signaling pathways and immune responses modulated by specific miRNA, as well as further improving our strategies for their safe and effective cellular delivery. While no clinical trials involving miRNA for gliomas have been instituted to date, recent research brings tremendous hope and potential for miRNA-based therapeutics as personalized therapeutic interventions for glioma patients.

## Figures and Tables

**Table 1:** Validated miRNAs involved in angiogenesis in gliomas.

miRNA	Regulation in Gliomas	Function	Targets	Validation	Reference
miR-7	Down-regulated	Inhibits angiogenesis	EGFR, IRS-1, IRS-2, FAK, OGR	Overexpression decreases cell proliferation and angiogenesis in U-87 cells in vitro and in tumor xenograft model	[43]
miR-296	Up-regulated	Promotes angiogenesis	HGS	Overexpression promotes angiogenesis in vitro and in a tumor xenograft model in vivo	[41]
miR-15b	Up-regulated	Inhibits angiogenesis	NRP-2	Overexpression reduces capillary tube formation in cultured endothelial cells	[46,47]
miR-93	Up-regulated	Promotes angiogenesis	Integrin B8	Overexpression increases cell migration and tube formation of co-cultured endothelial cells in vitro and in vivo	[48]

**Table 2:** Validated miRNAs involved in invasion in gliomas.

miRNA	Regulation in Gliomas	Function	Targets	Validation	Reference
miR-10b	Up-regulated	Promotes invasion	TP53, Pax6, Notch1, HOXD10	Overexpression increases invasion and its inhibition decreases invasion in vitro	[67]
miR-21	Up-regulated	Promotes invasion	PTEN, RECK, MARCKS	Inhibition elevates RECK and TIMP3 expression and decreases invasion in vitro and in vivo	[57]
miR-34a	Dow-regulated	Inhibits invasion	c-Met, Notch1/2, CDK6	Overexpression decreases invasion in glioma cells by targeting HGF/c-MET and Notch1/2 signaling; translated into clinical trial	[58]
miR-221/222	Up-regulated	Promotes invasion	p27, p57, EGFR/PTEN/AKT signaling, TIMP3, protein tyrosine phosphatase u	Overexpression increases invasion in multiple glioma cell lines	[68]
miR-124	Down-regulated	Inhibits invasion	IQGAP1	Overexpression in glioma cells inhibits cell migration and invasion	[69,70]
miR-181	Down-regulated	Inhibits invasion	IQGAP1, LAMC1, ITGB1	Overexpression decreases invasion in glioma cells	[71,72,73]
miR-451	Down-regulated	Inhibits invasion	BCL2, SALL4	Overexpression reduces invasion in glioma cells	[74,75]
miR-146	Down-regulated	Inhibits invasion	PI3K/AKT signaling, CAB39	Overexpression inhibits migration and invasion of glioma cells	[76]
miR-218	Down-regulated	Inhibits invasion	MMP16	Overexpression decreases invasion and inhibition increases invasion in glioma cells	[77]
miR-326	Down-regulated	Inhibits invasion	IKKB, MMP9	Overexpression inhibits invasion in glioma cells	[78]

**Table 3:** Validated miRNAs involved in cell metabolism in gliomas.

miRNA	Regulation in Gliomas	Function	Target	Validation	Reference
miR-153	Down-regulated	Restrained glutamine utilization and glutamate generation	Glutaminase	Overexpression decreased cell proliferation and glutamine utilization in glioma cells	[93]
miR-451	Up-regulated	Inhibition helps cancer cells escape metabolic stress	CAB39	Overexpression sensitized glioma cells to glucose deprivation	[74,94]
miR-326	Down-regulated	Decreases metabolism	PKM2, Notch signaling	Overexpression induces apoptosis and reduces metabolic activity in glioma cells	[78,95]
miR-106a	Down-regulated	SLC2A3	Glucose uptake	Inhibition decreases glucose uptake and proliferation in glioma cells	[96]
